# N_2_O Emissions from an Apple Orchard in the Coastal Area of Bohai Bay, China

**DOI:** 10.1155/2014/164732

**Published:** 2014-06-23

**Authors:** Baohua Xie, Junbao Yu, Xunhua Zheng, Fanzhu Qu, Yu Xu, Haitao Lin

**Affiliations:** ^1^Key Laboratory of Coastal Zone Environmental Processes and Ecological Remediation, Yantai Institute of Coastal Zone Research (YIC), Chinese Academy of Sciences (CAS), Shandong Provincial Key Laboratory of Coastal Zone Environmental Processes, YICCAS, Yantai Shandong 264003, China; ^2^State Key Laboratory of Atmospheric Boundary Layer Physics and Atmospheric Chemistry, Institute of Atmospheric Physics, Chinese Academy of Sciences, Beijing 100029, China; ^3^Agricultural Resources and Environment Institute, Shandong Academy of Agricultural Sciences, 202 Gongyebei Road, Jinan 250100, China

## Abstract

Using static chambers and gas chromatography, nitrous oxide (N_2_O) fluxes from an apple orchard soil in the Bohai Bay region of China were measured from February 2010 to February 2011. In this study, two nitrogen (N) fertilizer treatments were designed—without (CK) or with (SN) synthetic N fertilizers (800 kg N ha^−1^). The annual cumulative N_2_O emissions from CK and SN were 34.6 ± 3.0 (mean ± standard error) and 44.3 ± 6.0 kg N_2_O–N ha^−1^, respectively. Such high emissions resulted from the intensive N fertilization in the experimental and previous years. The direct emission factor (EF_d_) of N_2_O induced by the applied synthetic N fertilizers was 1.2%. The EF_d_ is within the range of previous studies carried out in other croplands, which suggests that it is reasonable to estimate regional N_2_O emissions from apple orchards using the EF_d_ obtained in other croplands. In addition, significant positive correlations existed between N_2_O fluxes and soil temperatures or soil dissolved organic carbon contents.

## 1. Introduction

Nitrous oxide (N_2_O) is an important atmospheric trace gas that contributes to global warming and stratosphere ozone depletion [1]. Mainly due to the use of nitrogen (N) fertilizer, agricultural soils are a major source of atmospheric N_2_O, releasing about 1.7–4.8 Tg N_2_O–N yr^−1^ [1]. Despite an increase in the number of N_2_O measurements from agricultural soils in recent years, there is still a great uncertainty in the current estimates of the total global N_2_O emission [2]. The uncertainty mainly originates from errors in measurements underlying emission factors and a lack of knowledge of emission processes [2, 3].

China currently consumes almost one-third of the world's N fertilizers (http://faostat.fao.org/default.aspx), in which the highest application rates of N fertilizers are in vegetable and fruit production [4, 5]. Many scientists have shown strong interest in the emissions of N_2_O from various Chinese croplands including the croplands of rice-wheat rotation, paddy rice, maize-wheat rotation, tea, and vegetables [5–12]. However, few investigations have been conducted in Chinese orchards, including apple, peach, and orange fields [13–15]. In other countries, studies on N_2_O emission from orchards are limited as well [2, 16]. The apple is the leading fruit in China and the area of apple orchards was 2.1 × 10^6^ ha in 2010 [17]. China has become the world's largest apple producing country, accounting for two-fifths and one-third of the total world apple acreage and yield, respectively (http://faostat.fao.org). The Bohai Bay region and the northwest Loess Plateau are the major apple producing areas and account for 86% and 90% of the Chinese apple acreage and yield, respectively [18]. Ju et al. [4] reported that the average application rate of N fertilizers in the apple orchards and greenhouse vegetables in the northeast of Shandong Province, located in the Bohai Bay region, was 882 and 3239 kg N ha^−1^ year^−1^, respectively. The highest rate of organic fertilizers in China is also applied in fruit production [19]. The direct emission factor (EF_d_) of N_2_O, that is, the ratio of the fertilizer induced N_2_O–N to the applied N fertilizers, is often used to estimate regional N_2_O emissions from agroecosystems. However, there are few studies which reported the emission factors obtained from orchards. The intensive N fertilization is likely to lead to a lot of N_2_O emissions [10]. Does the EF_d_ rise with the increased rate of N fertilization? Few researches were reported on it. Therefore, understanding the characteristics and quantifying the EF_d_ of N_2_O from intensively fertilized orchards will provide a scientific basis for better estimation of regional or global N_2_O emissions and developing mitigation options.

The objectives of this study were to determine the temporal variations of N_2_O fluxes and the EF_d_ of N_2_O induced by the applied synthetic N fertilizer in an apple orchard in the Bohai Bay region and to investigate the relationships between N_2_O fluxes and environmental factors, such as soil temperature, soil moisture, soil carbon, and nitrogen contents.

## 2. Materials and Methods

### 2.1. Site Description

The experimental site is located in the suburb (37°49′N, 120°45′E) of Penglai County in Shandong Province, neighboring the Bohai Bay. The investigated area is representative of the major apple production areas in China. This region displays a warm temperate continental monsoon climate. The annual precipitation is 664 mm. The annual mean air temperature is 11.9°C, and the annual frost-free period is approximately 206 days. The soil at the experimental site is Argosols (Cooperative Research Group on Chinese Soil Taxonomy, 2001) with 7.0% clay (<0.002 mm), 39.6% silt (0.002–0.02 mm), and 53.4% sand (0.02–2 mm). Other soil properties of the soil sample before the experiment beginning at a 0–20 cm depth are as follows: bulk density 1.39 g cm^−3^, soil organic carbon (SOC) 10.0 g kg^−1^, total nitrogen 1.2 g kg^−1^, total phosphorous 1.9 g kg^−1^, total potassium 15.8 g kg^−1^, available phosphorous 50.7 mg kg^−1^, available potassium 155.2 mg kg^−1^, and pH 6.7 (water).

### 2.2. Experiment Design

The experiment was performed in an apple orchard from February 2010 to February 2011. The apple orchard, which was converted from the former winter wheat field in 2002, was dominated by Fuji apple trees. The plant density is 670 plants ha^−1^, and the apples are usually harvested in mid-October. In the study, two N fertilization treatments, that is, without and with the addition of synthetic N fertilizer (hereinafter referred to as CK and SN, resp.), were applied. Both treatments were replicated three times. During 2010, urea (800 kg N ha^−1^), calcium biphosphate (175 kg P ha^−1^), and potassium sulfate (664 kg K ha^−1^) were applied in SN treatment, including the basal fertilization in early April and two dressing fertilizations in late June and mid-August (Table 1). The CK treatment was added with the same rates of P and K as SN but none synthetic N fertilizers. Several pesticides and fungicides were foliar sprayed two or three times per year in both SN and CK to prevent pests and diseases. Although the application rate of N fertilizers was very high, it was representative in Shandong Province. Ju et al. [4] reported that the average application rate of N fertilizers in the apple orchards in the northeast of Shandong Province was 882 kg N ha^−1^.

**Figure 1:**
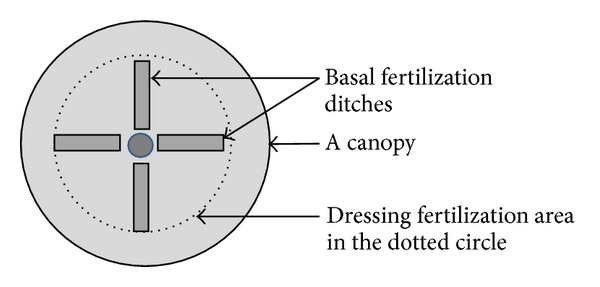
The diagram of fertilization area in the canopy projection area of an apple tree.

On April 2, 2010, the basal fertilizers were buried in ditches (length ∗ breadth ∗ depth = 1 ∗ 0.3 ∗ 0.2 m). Four ditches evenly scattered around an apple tree. The dressing fertilizations were evenly broadcasted on the ground under the trees one day after a rainfall or irrigation event in June and August 2010. The fertilization area for a tree is smaller than its canopy area.  Figure 1 showed the fertilization area for an apple tree. Both the basal and dressing fertilizers were applied inside the dotted circle of Figure 1. Due to the specific fertilization mode in the orchard, we separated a canopy projection area into two subplots, that is, inside and outside the dotted circle of Figure 1 (hereinafter referred to as site A and site B). Thus, SN and CK plots were separated to SN-A, SN-B, CK-A, and CK-B subplots for the sampling of gas and soil. The area ratio of sites A and B was 0.38.

### 2.3. Measurement of N_2_O Flux

The measurements of the N_2_O exchange flux* in situ* were performed from February 2010 to February 2011. Both of the CK and SN treatments were randomly set up with three replicates, and each replicate was separated to site A and site B subplots. The fluxes were generally measured once every 3-4 days, using static opaque chamber method in combination with gas chromatography techniques [21]. The sampling frequency was doubled in one week after a fertilization event and was reduced to once a week in cold winter.

For the sampling of N_2_O gas, a 0.25 m^2^ stainless-steel frame was permanently installed in the soil. A 0.5 m high, gas-tight chamber with water seals was temporarily mounted on the frame when the sampling occurred. The sampling process was completed between 09:00 and 11:00. Five gas samples were taken with 60 mL plastic syringes at 6 min intervals. Immediately after taking the fifth gas sample, the chamber was removed from the frame. Within at most 10 hours after sampling, the gas samples in the syringes were analyzed using a gas chromatograph with an electron capture detector (ECD) [21].

The nitrous oxide flux was calculated by the rate of the change in the N_2_O concentration in the chamber, estimated as the slope of linear regression between N_2_O concentration and time. The data on air pressure and chamber headspace air temperature were used to correct the N_2_O density at 273 K and 1,013 hPa to the actual headspace air conditions. Flux rates were discarded if the coefficient of determination (*r* ^2^) was less than 0.85. Single flux from the measurement between 9:00 and 11:00 was regarded as the daily mean flux and directly extrapolated to the cumulative emission for the observational period [22, 23]. The annual cumulative N_2_O emissions were calculated by linear interpolations between adjacent observations. The cumulative N_2_O emission from SN or CK treatment was determined by weighting the emissions from sites A and B on the basis of their area ratio of 0.38.

### 2.4. Auxiliary Measurements

Soil temperature, WFPS, inorganic nitrogen (IN, including NO_3_^−^– and NH_4_^+^–N), and dissolved organic carbon (DOC) were measured to determine the main factors that influence the emission of N_2_O.

Daily precipitation and air temperature were observed by a nearby automatic climate station. Soil temperatures and volumetric moisture at 10 cm depth were recorded automatically once an hour, though only the daily averages are reported here. Soil volumetric moisture values, which were measured with FDR sensor, were converted into values of water-filled pore space (%WFPS) by the following formula:

(1) $$\begin{array}{c} \%\text{WFPS}=\frac{\theta }{1-\text{BD}/\text{PD}}, \end{array}$$

where *θ* is the volumetric soil water content (%), BD is the bulk density (g m^−3^), and PD is the particle density constant (2.65 g m^−3^).

The surface soil (0–10 cm) was sampled biweekly using a 2 cm diameter gauge auger. At each sampling date, one sample, containing three bulked subsamples, was collected from each subplot. To measure NO_3_^−^ and NH_4_^+^ contents, fresh soil samples were extracted with a KCl solution (2 mol, soil : solution = 1 : 10) by shaking for 1 h. The extracts were analyzed with a continuous flow analyzer (Seal Bran-Lubbe AA3, Germany). Soil samples were extracted with deionized water (soil : water = 1 : 4) and the extracts were immediately analyzed for DOC with a total organic carbon analyzer (Shimadzu TOC-VCPH, Japan).

### 2.5. Statistical Analysis

In order to examine the relationships between the measured N_2_O fluxes and environmental parameters, exponential and linear regression analyses were performed. Differences in IN and DOC concentrations and N_2_O emissions among SN-A, SN-B, CK-A, and CK-B were determined using one-way ANOVA.

## 3. Results and Discussion

### 3.1. Environmental Conditions and Soil Parameters

**Figure 2:**
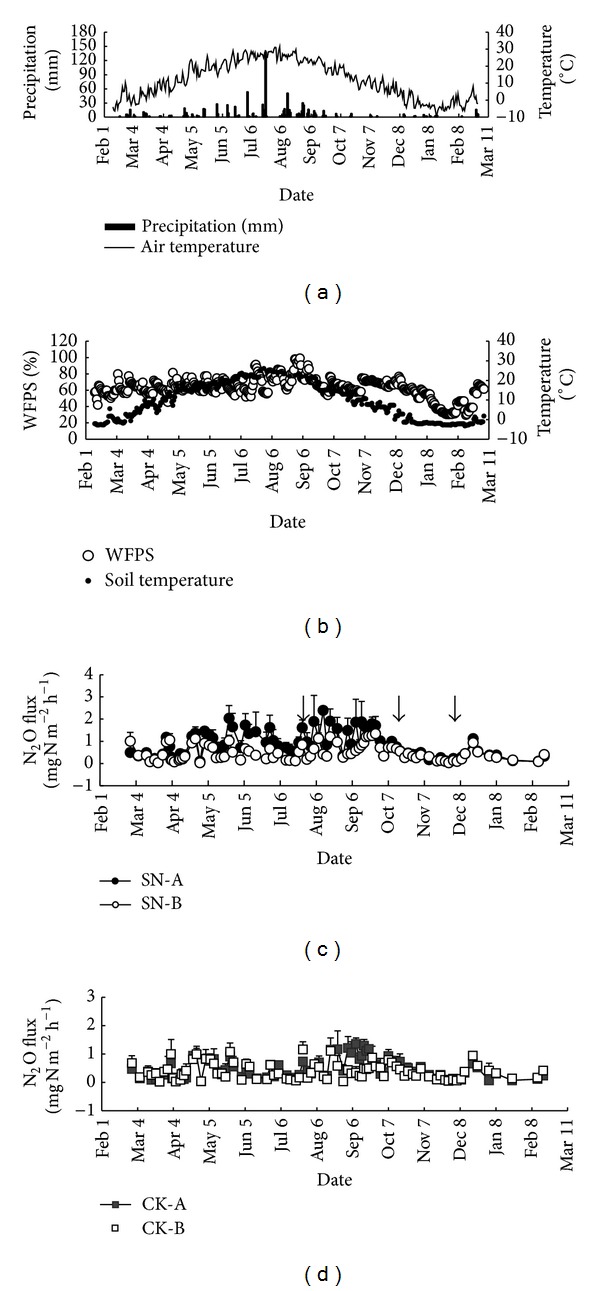
(a) Daily precipitation and average air temperature, (b) soil moisture (WFPS, i.e., the water-filled pore space) and soil temperature at 10 cm depth in site A (under the middle of an apple tree canopy), (c) N_2_O fluxes from SN, and (d) N_2_O fluxes from CK. The N_2_O data are the means and standard errors (the vertical bars) of three replicates. SN-A means site A (the fertilization area in an apple tree canopy projection area) in SN treatment. SN-B means site B (the unfertilization area in an apple tree canopy projection area) in SN treatment. CK-A and CK-B mean sites A and B in CK treatment, respectively. The downward arrows indicate the time of fertilization.

The annual average air temperature was 12.0°C and the total precipitation was 752 mm (Figure 2(a)).  Figure 2(b) showed soil temperature and WFPS at 10 cm depth. The variation pattern of soil temperatures was similar to that of air temperatures. Soil WFPS was 62% on average.

Table 2 listed the averages of soil DOC and IN concentrations during the experimental period. Both DOC and IN concentrations in SN-A were significantly higher than those in the other subplots. The average DOC concentration in SN-A was 153.9 mg kg^−1^, which was higher than that in SN-B, CK-A, and CK-B by 6% (*P* < 0.01), 9% (*P* < 0.01), and 27% (*P* < 0.01), respectively. The difference in DOC concentration in SN-B and CK-B was also significant (*P* < 0.05).

The average NO_3_^−^–N concentration in SN-A was 73.7 mg kg^−1^, which was higher than that in SN-B, CK-A, and CK-B by 148% (*P* < 0.01), 74% (*P* < 0.01), and 206% (*P* < 0.01), respectively. The average NH_4_^+^–N concentration in SN-A was 113.6 mg kg^−1^, which was higher than that in SN-B, CK-A, and CK-B by 963% (*P* < 0.01), 171% (*P* < 0.01), and 725% (*P* < 0.01), respectively. Due to the previous N fertilizations in CK-A in the previous years before the experiment, the concentrations of both NO_3_^−^–N and NH_4_^+^–N in CK-A were significantly higher than those in CK-B (*P* < 0.01).

### 3.2. N_2_O Fluxes and Annual Cumulative Emissions

The seasonal variation of N_2_O fluxes in SN-A was very large, followed by SN-B, CK-A, and CK-B in sequence (Figures 2(c) and 2(d)). In the fall and winter, N_2_O fluxes were low and varied only slightly in the four types of subplots. From May to October, N_2_O fluxes in SN-A were remarkably higher than those in the other subplots. Immediately after a synthetic N fertilization event, N_2_O fluxes usually increased temporarily in SN-A. The flux range in all the subplots was from 0.02 to 2.39 mg N_2_O–N m^−2^ h^−1^. The annual average N_2_O flux in SN-A was 0.83 mg N_2_O–N m^−2^ h^−1^, significantly higher than that in SN-B, CK-A, and CK-B by 79% (*P* < 0.05), 70% (*P* < 0.05), and 113% (*P* < 0.01), respectively.

Table 3 presented the annual cumulative N_2_O emissions. Mainly due to the application of synthetic N fertilizers, the annual cumulative N_2_O emissions in SN-A, 63.9 kg N_2_O–N ha^−1^, were higher than those in SN-B, CK-A, and CK-B by 74% (*P* < 0.01), 77% (*P* < 0.05), and 87% (*P* < 0.01), respectively. The weighed annual N_2_O emissions were 44.3 ± 6.0 and 34.6 ± 3.0 kg N_2_O–N ha^−1^ from SN and CK, respectively. The EF_d_ of N_2_O induced by the applied synthetic N fertilizer was 1.2 ± 0.4%.

### 3.3. Relationships between N_2_O Fluxes and Soil Temperature, WFPS, DOC, and IN

Because N fertilizers were added only to site A, we analyzed the relationships between N_2_O fluxes and soil parameters on sites A and B, respectively. Except for CK-B, N_2_O fluxes exhibited a significant exponential correlation with soil temperatures (*n* = 85, *P* < 0.01) and the determination coefficient (*R* ^2^) in site A was higher than that in site B (Table 4), which was in good agreement with the results from other studies [14, 15, 20]. Although a large number of previous studies suggested that N_2_O emission was significantly correlated with WFPS [20, 24, 25], there was no significant correlation between N_2_O fluxes and soil WFPS in this study. The possible reason is that the specific farm management such as fertilization and irrigation mode may conceal the relationship between N_2_O emissions and soil WFPS. Lin et al. [14] reported that N_2_O emissions from an orange orchard were positively correlated with soil temperature but not correlated with WFPS. Pang et al. [15] also reported a similar finding in a study which was performed in an apple orchard.

N_2_O emissions increase with the increase of SOC contents [24]. N_2_O fluxes exhibited a significant positive correlation with soil DOC in both CK and SN treatments (Table 4). The determination coefficients in CK were higher than those in SN, in which DOC contents were higher than those in CK (Table 2). The results suggested that the effect of DOC on N_2_O emissions might become weak when DOC content was higher than some threshold value.

**Figure 3:**
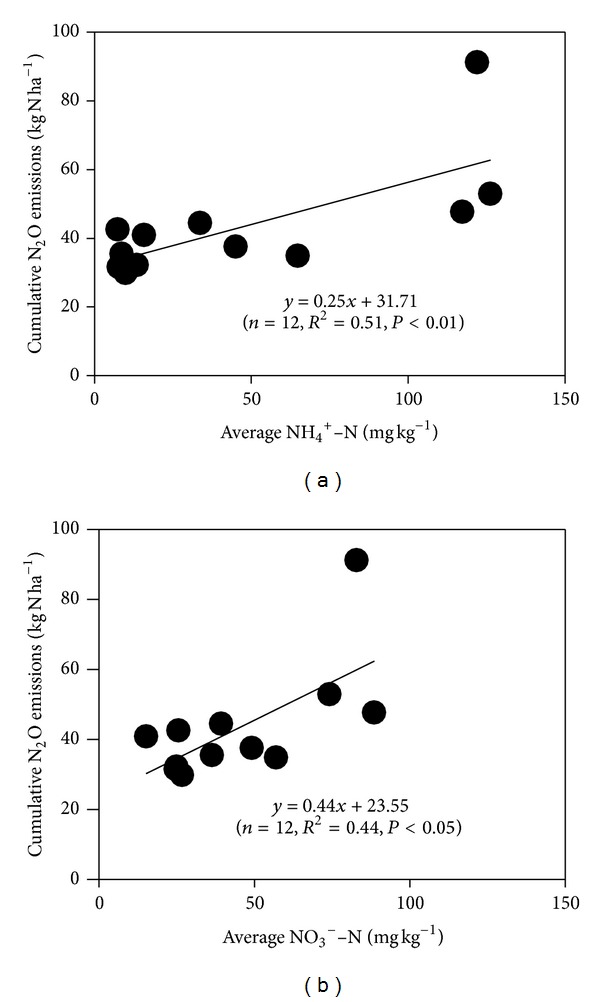
Relationships between the annual cumulative N_2_O emissions and the annual average (a) NH_4_^+^–N and (b) NO_3_^−^–N contents. The given data are obtained from all of the 12 subplots.

Nitrous oxide is mainly produced in soil by nitrification and denitrification, which are particularly controlled by the amount of ammonium and nitrate [26, 27]. Although the seasonal variations of N_2_O fluxes were not significantly correlated with the dynamics of soil NO_3_^−^–N or NH_4_^+^–N contents, the annual cumulative N_2_O emissions in all of the subplots exhibited significant positive correlations with the annual average contents of soil NO_3_^−^–N and NH_4_^+^–N (Figure 3). The results suggested that the contents of soil inorganic N should be one of the major reasons controlling the annual N_2_O emissions from various subplots.

### 3.4. Comparison with Other Studies on N_2_O Emissions from Orchard Soils

To date, there were few reports on N_2_O emissions from orchard soils.  Table 5 listed soil properties, annual N fertilizations, and N_2_O emissions from some orchard soils. Pang et al. [15] reported that N_2_O emission from an apple orchard was 2.05 kg N_2_O–N ha^−1^ year^−1^. Lin et al. [14] found that N_2_O emissions were 1.55–2.03 kg N_2_O–N ha^−1^ year^−1^ from orange orchard soils. Lin et al. [13] reported that the annual cumulative N_2_O emission was 1.4 kg N_2_O–N ha^−1^ from a peach orchard. Liu et al. [20] reported a high N_2_O emission of 8.64 kg N_2_O–N ha^−1^ from a longan orchard without fertilization. The annual cumulative N_2_O emissions in these previous studies were only 0.1%–24.9% of the emissions from SN in the present study. There may be two major reasons leading to such a great distinction. The first one is the enormous difference in the nitrogen fertilizer application rates; the rates in the above literatures were 0–597 kg N ha^−1^, only 0%–74.6% of that in the present study. Many previous studies suggested that N_2_O emissions from fertilized soils were positively correlated with the nitrogen fertilization rates [28–30]. N_2_O emissions were found to be very high in cultivated soils which were incorporated with high amount of N fertilizers. For example, Mei et al. [5] reported that the annual cumulative N_2_O emissions from the vegetable fields incorporated with N fertilization rates of 1195 ± 63 kg N ha^−1^ year^−1^ were 36.7 ± 14 kg N_2_O–N ha^−1^. The second reason is the very low measurement frequencies in the previous studies, that is, biweekly [13, 15] or even monthly [14] interval, which probably led to missing some peak fluxes of N_2_O in case of significant changes in soil moistures or nitrogen fertilization events [5, 8, 16, 31]. Liu et al. [20] measured N_2_O fluxes at the same frequency as this study, that is, twice a week, and found that N_2_O emission from a longan orchard without fertilization was 8.64 kg N_2_O–N ha^−1^, which was severalfold of those values obtained from much lower frequency of measurements in orchards [13–15].

It should be noted that N_2_O emission from CK was as high as 33.1 ± 3.8 kg N_2_O–N ha^−1^. Nitrous oxide is mainly produced in soil by nitrification and denitrification, and N_2_O emissions were particularly controlled by the amount of ammonium and nitrate [26, 27]. The CK treatment received a large amount of N fertilizers in previous years before the experiment, resulting in high amounts of both NO_3_^−^–N and NH_4_^+^–N in the experimental period (Table 2). In CK-A, the content of soil NO_3_^−^–N and NH_4_^+^–N was up to 42.4 ± 12.4 and 42.0 ± 14.4 mg kg^−1^, respectively. Even in CK-B, the content of soil NO_3_^−^–N and NH_4_^+^–N was up to 24.1 ± 9.2 and 13.8 ± 7.5 mg kg^−1^, respectively. The high contents of IN in CK probably resulted in high emissions of N_2_O. Liu et al. [20] reported that N_2_O emission was up to 8.64 kg N_2_O–N ha^−1^ from a none-N fertilization longan orchard which was fertilized before the experimental year.

### 3.5. Emission Factor of N_2_O Induced by the Applied Synthetic N Fertilizer

Based on the available site-scale data sets of N_2_O emissions in annual paddy rice-wheat rotation systems, Zou et al. [28] reported that the EF_d_ of N_2_O induced by the applied synthetic fertilizer averaged 1.02% for the rice season, 1.65% for the wheat season, and 1.25% for the annual season. Xiong et al. [32] reported that the EF_d_ of N_2_O was 0.73% from a greenhouse vegetable field which was added with N fertilizers at the rate of 1636 kg N ha^−1^ year^−1^. Bouwman et al. [33] reported that the EF_d_ of N_2_O induced by the applied synthetic N fertilizer averaged 1.0% and the value was renewed as 0.91% [2]. Due to the high amount of N fertilizer application, the annual cumulative emission of N_2_O was very high in the present study, though the EF_d_ was 1.2%, close to the previous studies. The result suggests that the EF_d_ of the apple orchard is close to other croplands, and it may be reasonable to estimate N_2_O emissions from the apple orchard soil using the EF_d_ obtained from other croplands.

## 4. Conclusions

Mainly due to the high application rate of N fertilizers, the annual N_2_O emission from an apple orchard in the Bohai Bay region, China, was up to 44.3 ± 6.0 kg N_2_O–N ha^−1^, which indicated that the apple orchard is an important source of atmospheric N_2_O. Apple production must be taken into account when estimating N_2_O emissions from agroecosystems. The EF_d_ of N_2_O induced by the applied synthetic N fertilizer in the present study was 1.2%, which was within the range of EF_d_ obtained in other croplands. Thus, it may be reasonable to estimate N_2_O emissions from apple orchard soils using the EF_d_ obtained in other croplands.

## Figures and Tables

**Table 1:** Details of fertilization events in the apple orchard.

Fertilizers^a^	Total amount	Date of fertilizations		
	(kg ha^−1^)	2010-4-2	2010-6-24	2010-8-16
N	800	400	120	280
P	175	175	0	0
K	664	332	0	332

^a^Synthetic fertilizers of N, P, and K were urea, calcium biphosphate, and potassium sulfate, respectively.

**Table 2:** Average concentrations (mean ± SE) of soil DOC (mg C kg^−1^) and IN (mg N kg^−1^) during the experimental period.

Items	N treatment	Site A	Site B	Weighted average
DOC	SN	153.9 ± 13.0	145.4 ± 12.3	151.4 ± 12.8
	CK	140.9 ± 16.7	121.0 ± 17.2	135.0 ± 16.8

NO_3_^−^–N	SN	73.7 ± 14.1	29.7 ± 9.0	60.5 ± 12.6
	CK	42.4 ± 12.4	24.1 ± 9.2	36.9 ± 12.9

NH_4_^+^–N	SN	113.6 ± 22.8	10.7 ± 4.2	82.8 ± 17.2
	CK	42.0 ± 14.4	13.8 ± 7.5	33.5 ± 12.3

SN means the synthetic N fertilizer treatment. CK means the treatment without synthetic N fertilizer. Site A means the fertilization area in an apple tree canopy projection area. Site B means the nonfertilization area in an apple tree canopy projection area. The weighted averages were calculated from the emissions from site A and site B according to their area ratio.

**Table 3:** Annual cumulative emissions of N_2_O (mean ± SE, kg N_2_O–N ha^−1^).

N treatment	Site A	Site B	Weighted average
SN	63.9 ± 13.7	36.7 ± 3.1	44.3 ± 6.0
CK	36.1 ± 1.9	34.1 ± 3.4	34.6 ± 3.0

The meanings of SN, CK, site A, and site B can be found in the text and the footnote in Table 2. The weighted averages were calculated from the emissions of site A and site B according to their area ratio.

**Table 4:** Summary of regression analysis.

N treatment	Variables	Regressions	*R* ^2^	*n*	*P*
CK-A	ST	*y* = 0.167*e*0.05*x*	0.25	85	<0.01
SN-A	ST	*y* = 0.229*e*0.07*x*	0.49	85	<0.01
SN-B	ST	*y* = 0.209*e*0.07*x*	0.15	85	<0.01
CK-A	DOC	*y* = 0.004*x* − 0.202	0.23	26	<0.05
SN-A	DOC	*y* = 0.005*x* − 0.139	0.08	26	<0.1

The variable *y* means N_2_O fluxes. The variable *x* means the environmental factor including soil temperature and soil DOC.

**Table 5:** Soil properties, annual N fertilizations, and N_2_O emissions from orchard soils.

Land use	TN (g kg^−1^)	SOC (g kg^−1^)	C/N ratio	NH_4_^+^–N (mg kg^−1^)	NO_3_^−^–N (mg kg^−1^)	N fertilizer (kg N ha^−1^)	N_2_O emissions (kg N ha^−1^)	References
Apple	1.42	10.0	6.9	82.8	60.5	800	44.3	This study
Apple	1.36	10.0	7.4	33.5	36.9	0^a^	34.6	This study
Apple	1.00	6.3				312	3.2	[15]
Orange	1.10			7.4	27.1	597	2.03	[14]
Orange	1.06			8.5	65.2	579	1.55	[14]
Peach	1.38	6.3				210	1.4	[13]
Longan	1.49	13.4	10.0			0^a^	8.64	[20]

^a^Nitrogen fertilizers were applied before the experimental years (Liu et al. [20] and this study).
